# Advances in the mechanism of metformin with wide-ranging effects on regulation of the intestinal microbiota

**DOI:** 10.3389/fmicb.2024.1396031

**Published:** 2024-05-24

**Authors:** Yue Wang, Xianxian Jia, Bin Cong

**Affiliations:** ^1^College of Forensic Medicine, Hebei Key Laboratory of Forensic Medicine, Hebei Medical University, Shijiazhuang, China; ^2^Research Unit of Digestive Tract Microecosystem Pharmacology and Toxicology, Chinese Academy of Medical Sciences, Beijing, China; ^3^Department of Pathogen Biology, Institute of Basic Medicine, Hebei Medical University, Shijiazhuang, Hebei, China

**Keywords:** metformin, intestinal microbiota, gastrointestinal mucosal barrier, SCFAs, bile acid metabolism

## Abstract

Metformin is of great focus because of its high safety, low side effects, and various effects other than lowering blood sugar, such as anti-inflammation, anti-tumor, and anti-aging. Studies have shown that metformin has a modulating effect on the composition and function of the intestinal microbiota other than acting on the liver. However, the composition of microbiota is complex and varies to some extent between species and individuals, and the experimental design of each study is also different. Multiple factors present a major obstacle to better comprehending the effects of metformin on the gut microbiota. This paper reviews the regulatory effects of metformin on the gut microbiota, such as increasing the abundance of genus *Akkermansia*, enriching short-chain fatty acids (SCFAs)-producing bacterial genus, and regulating gene expression of certain genera. The intestinal microbiota is a large and vital ecosystem in the human body and is considered to be the equivalent of an “organ” of the human body, which is highly relevant to human health and disease status. There are a lot of evidences that the gut microbiota is responsible for metformin’s widespread effects. However, there are only a few systematic studies on this mechanism, and the specific mechanism is still unclear. This paper aims to summarize the possible mechanism of metformin in relation to gut microbiota.

## 1 Introduction

Metformin is a synthetic derivative of guanidine derived from the guanidine alkaloid of the plant named *Galega officinalis* L. with significant hypoglycemic effects (Ursini et al., 2018), which was first successfully synthesized by two Irish scientists, Werner and Bell (1922). Since its clinical application in 1957, metformin’s cornerstone status has remained consistent and it is still the first-line preferred treatment for type 2 diabetes mellitus (T2DM) side by side with lifestyle (Montvida et al., 2018; Nicolucci et al., 2019; Ahmad et al., 2020). T2DM is a chronic, low-grade inflammatory disease characterized by elevated blood glucose and is regulated by a combination of genetics and environment. The incidence of T2DM continues to rise worldwide due to people’s bad dietary habits, the decrease in exercise and unhealthy lifestyles. According to the International Diabetes Federation, 1 in 8 adults, about 783 million people, will live with diabetes by 2045, and more than 90% of them will have T2DM (The Idf Diabetes Atlas, 2021). Metformin is widely used because of its low cost, good efficacy and few adverse reactions (Lancet, 1998), but its exact mechanism of action still remains partly unclear.

Now the confirmed mechanism of metformin’s hypoglycemic action is to inhibit hepatic gluconeogenesis and reduce hepatic glucose output through both AMPK-dependent (Song et al., 2001; Shaw et al., 2005; Miller et al., 2013) and AMPK-independent (Madiraju et al., 2014, 2018) pathways. At the same time, metformin also promotes the uptake and utilization of glucose by peripheral tissues. Extensive scientific evidences have also shown that in the gut, metformin can also increase the secretion of glucagon-like peptide-1 (GLP-1) by L cells (Kim et al., 2014; Napolitano et al., 2014; Duca et al., 2015), thereby improving blood glucose homeostasis and reducing the secretion of lipids by intestinal epithelial cells. Therefore, metformin can not only regulate glucose metabolism, but also regulate lipid metabolism. This feature makes it very suitable for obese patients with T2DM. In addition, metformin was also found to have anti-tumor effects.

The process of metformin exerting its extensive effects cannot be separated from the regulation of gut microbiota. According research (Koropatkin and Martens, 2017), when metformin is administered orally, its bioavailability is about 50%, and it is higher compared to intravenous administration. Its action mainly occurs in the intestine, suggesting that intestinal microbiota may be another target of metformin (Wang J. H. et al., 2018). With the widespread use of metformin in clinical practice, the research on it has become increasingly advanced. There have been substantial evidences that metformin has a regulatory effect on the gut microbiota (Zhang et al., 2015; Elbere et al., 2018; Harsch and Konturek, 2018; Brandt et al., 2019), and the process by which metformin exerts its broad effects is also closely correlated with the gut microbiota. Even though this correlation has been verified, its specific mechanisms are not fully understood. In this paper, we comprehensively review the progress of its research.

## 2 The main process of metformin in the human body

**Figure 1:**
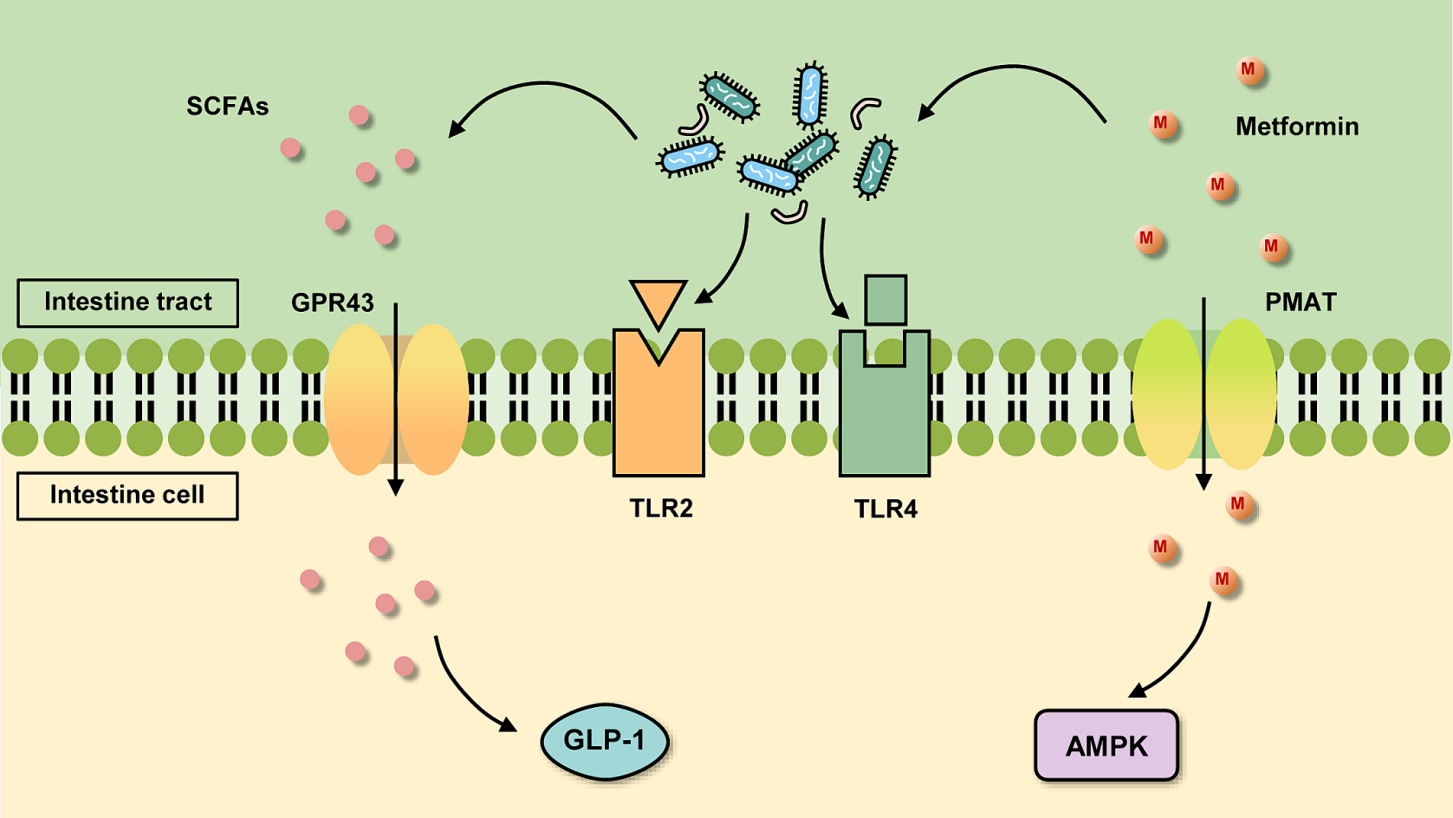
Basic mechanism of metformin action in the intestine. On the one hand, metformin is absorbed by intestinal cells through transporters such as plasma monoamine transporter protein (PMAT) and organic cation transporter protein 3 (OCT3), which activate the AMPK pathway. On the other hand, it can act in the gut on the gut microbiota, which changes and acts on certain receptors or produces certain metabolites for further regulation.

After oral administration of metformin, it enters the gastrointestinal (GI) tract and is absorbed by the intestinal epithelial cells of the upper small intestine. This absorption in the stomach and large intestine is almost negligible (Graham et al., 2011). Because the hydrophilicity of the drug inhibits its transport process across cell membranes, metformin must rely on transporters to actively transport in and out of cells (Hardie et al., 2012). Intestinal epithelial cells take up metformin through transporters located on the inner surface of the intestinal epithelium such as plasma monoamine transporter protein (PMAT) and organic cation transporter protein 3 (OCT3) (Figure 1). However, some studies have found that metformin is also taken up in the intestine by passive diffusion. But there is no consensus on whether this type of translocation occurs via paracellular or transcellular pathways (Shirasaka et al., 2022). The drug then leaves the intestinal epithelium via organic cation transporter protein 1 (OCT1) and is delivered to the liver via the portal vein. Metformin enters the liver via OCT1 and OCT3, where it suppresses gluconeogenesis. Multi-specificity is one of the most distinguishing features of OCT1 (Meyer and Tzvetkov, 2021). Drugs are not metabolized by the liver, but multidrug and toxin extrusion protein 1 (MATE1) expressed in hepatocytes is involved in the clearance of drugs, which is transported to the kidney through bile or through blood (Gong et al., 2012). Metformin then enters the renal epithelium via organic cation transporter protein 2 (OCT2). The drug is then secreted by MATE1 and MATE2 of the kidney in an unaltered form and is cleared through the urine.

It is well known that the pharmacodynamic property of metformin is anti-diabetic, but there is growing evidence that it has a number of extra-hypoglycemic effects. Firstly, metformin has been shown to improve lipid metabolism (He, 2020) and therefore has a favorable effect on body weight (Day et al., 2019; Zhang et al., 2023), blood lipids (Wulffelé et al., 2004), and cardiovascular risk (Després, 2003) associated with T2DM. Secondly, the role of metformin that has received the most attention is its effect on tumor tissue, not only in terms of treatment, but also in terms of prevention (Coyle et al., 2016; Cejuela et al., 2022). In addition, many studies have emphasized the anti-inflammatory (Pålsson-McDermott and O’Neill, 2020) and anti-aging (Soukas et al., 2019) effects of metformin. Current research done on the potential beneficial effects of metformin has extended to treating diabetic nephropathy (Han et al., 2021), metabolic syndrome (Ladeiras-Lopes et al., 2015), and polycystic ovary syndrome (Peng et al., 2023). There are even recent studies that have found metformin to have protective and therapeutic effects against COVID-19 (Bramante et al., 2021, 2023).

## 3 Overview of the gut microbiota

### 3.1 Structure of the gut microbiota

The gut microbiota is a fairly complex ecosystem. Luckey (1972) found in 1972 that the number of human intestinal microbiota can reach 10^14^ species, and this data has been accepted and quoted by most scholars after publication. The amount of genetic material in the intestinal microbiota exceeds the human genome by more than 100 times (Bäckhed et al., 2005). The intestinal microbial community is diverse and dominated by five main bacterial phyla: Bacteroidetes, Firmicutes, Actinobacteria, Proteobacteria and Verrucomicrobia. Among these five phyla, the phylum Bacteroidetes and the phylum Firmicutes accounted for more than 90% of the total population. Most of the bacteria under the phylum Bacteroidetes belong to the genera *Bacteroides* and *Prevotella*. And bacteria under the phylum Firmicutes that dominate the gut microbiota include the genera *Clostridium*, *Eubacterium* and *Ruminococcus* (Eckburg et al., 2005). The proportion of various microbiota is different depending on the site of the GI tract. The upper GI tract was mainly enriched with Firmicutes, Proteobacteria and *Lactobacillaceae*, while the microbiota of lower GI tract was mainly composed of Bacteroidetes, Firmicutes, and *A.muciniphila* (Scheithauer et al., 2016; Meijnikman et al., 2018). In addition to taxonomic classification, the human microbiota can be categorized into three different enterotypes: *Bacteroides*, *Prevotella* or *Ruminococcus*. *Bacteroides*-dominated enterotype is characterized by saccharolytic and proteolytic activities involved in synthesis of riboflavin, biotin, ascorbate and pantothenate. The enterotype of *Prevotella*-dominated plays the role of mucin glycoprotein degraders involved in the synthesis of thiamine and folate. The last enterotype is dominated by *Ruminococcus*, characterized by membrane transportation of sugars and mucin degrading activities (Arumugam et al., 2011). A study from Taiwan categorized enterotypes into *Bacteroides*, *Prevotella* and *Enterobacteriaceae* and claimed that *Enterobacteriaceae* may be a new subtype in Asian populations (Liang et al., 2017). However, the concept of enterotypes has been debated due to the high degree of variability observed in the gut microbiota between individuals and the fact that many of the data show that there are not three completely discrete clusters (Knights et al., 2014). It is indisputable that regardless of enterotypes, some members of the microbial population act as a “core microbiota,” while others are more of a “flexible pool.” The “core microbiota” is composed of host-adapted microbes reproducibly included in the gut microbiota made up of different environmental combinations and determined by genetic factors. While the “flexible pool” is usually obtained from water, food and various components of the environment, which contributes to the adaptation of the host (Shapira, 2016).

### 3.2 Gut microbiota vs. disease

Human cells coexist with bacteria for a long time, and there is a complex process of material and energy exchange between them. Intestinal microbiota plays a crucial role in many key metabolic processes of human body, such as SCFAs, amino acids, bile acids and vitamin synthesis (Montandon and Jornayvaz, 2017). There is no doubt that the gut microbiome is extremely relevant to human health and disease status. In many disease states, such as metabolic syndrome, inflammatory bowel disease (IBD), cardiovascular diseases, malignant tumors, etc., the structure and function of intestinal microbiota are obviously different from those of healthy bowel microbiota (Tilg and Moschen, 2014; Shin et al., 2017; Harsch and Konturek, 2018; Eibl and Rozengurt, 2019). Regulating the structure and function of intestinal microflora can improve the disease status of the body to a certain extent. So, targeting the gut microbiome may provide new therapeutic thoughts for some intractable diseases. In particular, the role of gut microbiota in T2DM and obesity has attracted the attention of many researchers (Boulangé et al., 2016; Pedersen et al., 2016; Cani, 2018; Chobot et al., 2018; Zhao et al., 2018). Gut bacterial therapies that improve insulin sensitivity by altering the composition of the gut microbiota have become a new therapeutic modality.

Study has shown that changes in modern lifestyles have led to a decrease in the diversity of the gut microbiota in many developed populations (Clemente et al., 2015). This reduced diversity may promote the development of metabolic disorders. Individuals with low gut microbiota abundance have been found to be susceptible to obesity, insulin resistance and dyslipidemia (le Chatelier et al., 2013). *Lactobacillus*, *Prevotella*, *Bacteroides*, *Desulfovibrio*, and *Oxalobacter* spp. are decreasing in the gut microbiota of low-gene-count patients compared with high-gene-count patients. Functional changes in the microbiota of low-gene-count patients primarily consist of a decrease in butyrate-producing bacteria and an increase in the ratio of *Akkermansia* to *Ruminococcus gnavus*, which lead to decreased methane production potential, decreased hydrogen production potential, enhanced mucus degradation, and increased peroxidase activity (le Chatelier et al., 2013). The metabolic disturbances resulting from this imbalance of anti-inflammatory and pro-inflammatory bacterial species puts people at increased risk of suffering from T2DM (Ouchi et al., 2011). Approximately 86% of patients with T2DM are overweight or obese, and obesity is considered the greatest risk factor for T2DM (Daousi et al., 2006; Singer-Englar et al., 2019). A number of studies have reported significant changes in the gut microbiota of patients with obesity and T2DM compared to healthy adults (Qin et al., 2012; Karlsson et al., 2013; Tims et al., 2013; Forslund et al., 2015; Dao et al., 2016; le Roy et al., 2018), manifested in an overall increase in the abundance of *Bacteroides* and *Prevotella copri* as well as a decrease in the abundance of *Akkermansia*, *Roseburia* and *Ruminococcus*.

## 4 Regulation of intestinal microbiota by metformin

### 4.1 Overview

Metformin comes from *Galega officinalis* L., and later the function of lowering blood glucose has been found in animals, and more and more studies on its hypoglycemic effect have been conducted, and the effect has been furtherly confirmed. There has also been a great deal of research on its mechanisms. Stepensky et al. (2002) stated that metformin was better able to lower blood glucose by intestinal administration. Subsequently, researchers boldly hypothesized that metformin acted through the gut. Duca et al. (2015) found that the hypoglycemic effect of metformin was reduced by blocking the cAMP pathway in the gut, and speculated that the target of metformin was mainly in the gut. Buse et al. (2016) confirmed again that the gut is the main locus of hypoglycemic effects of metformin through comparable plasma levels of metformin. Meanwhile, due to its safety and few side effects, metformin has been widely studied, and it has been found that its effect is not only limited to reducing hyperglycemia, but also delaying the development of diabetes complications (Scheen and Paquot, 2013; Yaribeygi et al., 2019; American Diabetes Association, 2020). Therefore, people have found that it has many extra-hypoglycemic effects, such as anti-inflammatory, anti-tumor, anti-aging and so on. Among them, the anti-inflammatory effect has attracted the most attention, because in many diseased states, besides specific symptoms the body is generally in a state of chronic inflammation.

The mechanism of metformin has been studied endlessly, but the mechanism has not yet been fully clarified. In recent years, with advances in gene sequencing technology, more and more research has focused on the effects of metformin on the gut microbiota. It has been possible to confirm the association of metformin with certain intestinal microbiota. Most animal studies (Lee et al., 2018; Wang Z. et al., 2018; Zheng et al., 2018; Brandt et al., 2019; Ji et al., 2019; Ahmadi et al., 2020; Chung et al., 2020; Ryan et al., 2020) used C57BL/6 mice to construct a high-fat diet (HFD)-induced diabetes model followed by metformin treatment. Some studies have also used db/db mice (Chen et al., 2018; Zhang et al., 2019) or KKAy mice (Gao et al., 2018). Although the structure of the intestinal microbiota is very complex and the experimental design varies among studies, such as the different timing of HFD induction, distinctions in the duration of metformin treatment, and variations in mouse strains, but the impact of metformin on gut microbiota derived from these studies are nearly consistent. For example, the proportions of phyla Bacteroidetes (Gao et al., 2018; Ahmadi et al., 2020; Ryan et al., 2020) and Verrucomicrobia (Gao et al., 2018; Lee et al., 2018; Ryan et al., 2020) and genera *Akkermansia* (Chen et al., 2018; Gao et al., 2018; Lee et al., 2018; Zheng et al., 2018; Ji et al., 2019; Zhang et al., 2019; Ryan et al., 2020) and Bacteroides (Chen et al., 2018; Gao et al., 2018; Lee et al., 2018; Ryan et al., 2020) were significantly increased in the metformin treatment group. Similar results were also manifested in the rat model as in the mouse model (Zhang et al., 2015; Cui et al., 2019). Details are shown in Table 1.

**Table 1:** Changes in gut microbiota after administration of metformin therapy.

Research object	Increased gut bacteria	Decreased gut bacteria	References
HFD C57BL/6 mice	*Bacteroides, Akkermansia, Parabacteroides*	*Ruminococcus, Desulfovibrio, Dorea, Lachnoclostridium*	Ryan et al. (2020)
HFD C57BL/6 mice	*Akkermansia, Bacteroides, Butyricimonas, Verrucomicrobia, Parabacteroides*	*Firmicutes/Bacteroidetes ratio*	Lee et al. (2018)
db/db mice	*Akkermansia, Butyricimonas, Lactobacillus, Coprococcus, Ruminococcus*	*Prevotella, Proteus*	Zhang et al. (2019)
db/db mice	*Akkermansia, Bacteroides, Bacteroidales, Lactobacillus, Allobaculum*	*Enterococcus, Staphylococcus, Corynebacterium, Jeotgalicoccus, Aerococcus, Facklamia*	Chen et al. (2018)
KKAy mice	*Bacteroides, Akkermansia, Enterococcus, Proteus, Parasutterella, Escherichia/Shigella*	*Lactobacillus, Lachnoclostridium, Pseudomonas, Staphylococcus*	Gao et al. (2018)
HFD SD rats	*Roseburia, Akkermansia, Lactobacillus*	*Desulfovibrio, Lachnospiraceae NK4A136*	Cui et al. (2019)
HFD Wistar rats	*Akkermensia, Bacteroides, Butyricicoccus, Lactobacillus, Parasutterella, Klebsiella, Prevotella, Allobaculum*	*Clostridium XIVa, Clostridium XI, Flavonifractor, Roseburia, Lachnospiracea*	Zhang et al. (2015)
T2DM patients	*Akkermansia muciniphila, Pectobacterium, Pantoea, Serratia, Bacillus, Pseudomonas, Klebsiella, Enterobacter, Escherichia*	*Dethiosulfovibrio, Deferribacter, Pseudogulbenkiania, Pseudoflavonifractor, Subdoligranulum, Intestinibacter*	Wu et al. (2017)
T2DM patients	*Akkermansia muciniphila, Prevotella, Butyrivibrio, Bifidobacterium bifidum, Megasphaera*	*Clostridiaceae 02d06, Oscillospira, Barnesiellaceae*	de la Cuesta-Zuluaga et al. (2017)
T2DM patients			Forslund et al., 2015
Obesity patients	*Akkermansia, Klebsiella Clostridium XIVa, Clostridium XIVb, Escherichia/Shigella*	*Clostridium XI, Clostridium XVIII, Roseburia*	Hiel et al. (2020)
Healthy individuals	*Escherichia/Shigella*	*Intestinibacter*	Bryrup et al. (2019)

However, the results in humans are not entirely consistent with those in animals. This may be due to the significant differences in gut microbial diversity between humans and animals (Peng et al., 2013). Moreover, clinical trials in patients with diabetes are affected by many complex factors, such as diet, race, comorbidities, and drug combinations. An analysis of a T2DM cohort based on metagenomic sequencing and validated in an independent cohort found that metformin-treated patients had an increase in *Escherichia coli* and a decrease in *Intestinibacter*, and that the abundance of several intestinal microbial genera was more similar to that of normal control levels, particularly *Akkermansia* (Forslund et al., 2015). Moreover, A double-blind study of patients with T2DM treated with metformin led to significant changes in the relative abundance of more than 80 bacterial species compared with placebo after 4 months, in which an increase in *Escherichia* and *Akkermansia muciniphila* and a decrease in *Intestinibacter* were also observed, whereas most of the changes with placebo occurred in phylum Firmicutes and phylum Proteobacteria (Wu et al., 2017). Similar results were found in several studies on the effects of metformin on the gut microbiota of patients with obesity or T2DM (de la Cuesta-Zuluaga et al., 2017; Hiel et al., 2020), and even a study on metformin-induced changes in the gut microbiota of healthy young men likewise observed an increase in *Escherichia/Shigella* and a reduction in *Intestinibacter* (Bryrup et al., 2019). Details are shown in Table 1.

Nevertheless, the alterations in the gut microbiota of diabetic individuals by metformin treatment show consistent results in certain bacteria, in both humans and rodents. This was almost always demonstrated by enrichment of mucin-degrading *A. muciniphila* and SCFA-producing bacteria such as *Bifidobacterium bifidum* and *Butyrivibrio* (de la Cuesta-Zuluaga et al., 2017). However, this discrepancy between the human and animal studies suggests that we should still focus our researches in clinical setting and interpreting the mechanisms of action of the human gut microbiota. However, the reported results on the effect of metformin on human gut microbiota diversity are inconsistent. In patients with newly diagnosed T2DM, Tong et al. (2018) reported an increase in gut microbiota diversity with metformin based on Chao1 enrichment estimates, whereas Sun et al. (2018) observed a slight decrease in alpha diversity. In studies of healthy individuals, Bryrup et al. (2019) found no significant changes in gut microbial richness and diversity after metformin treatment. However, Elbere et al. (2018) reported a significant decrease in gut microbiota diversity after 24 h of metformin administration using the Shannon index. Overall, the therapy of metformin was associated with changes in the abundance of specific bacterial genera in the human gut microbiota, but the effects on gut microbiota diversity were variable, which highlights the need for further research to understand the potential mechanisms and clinical significance.

### 4.2 Regulation of *Akkermansia muciniphila* by metformin

*Akkermansia muciniphila*, a strictly anaerobic, endospore-free ovoid gut bacterium, is true of the phylum Verrucomicrobia (Derrien et al., 2004). It mainly colonizes the outer mucus layer of the GI tract, and uses the mucin of the GI tract as a carbon and nitrogen source to maintain its growth. The dynamic balance between the consumption of mucin and the production of mucin by goblet cells is obtained to maintain the stability of the mucus layer (Sicard et al., 2017). And the abundance of *A. muciniphila* is obviously reduced in patients with obesity, T2DM, and cardiovascular disease. At the same time, a large number of studies have shown that the content of mucin-degrading bacteria *A. muciniphila* in the intestinal microbiota is significantly increased after the administration of metformin (Hur and Lee, 2015; de la Cuesta-Zuluaga et al., 2017; Wu et al., 2017; Lee et al., 2018; Verdura et al., 2019), which can make it reach 20% of the total microbiome (de la Cuesta-Zuluaga et al., 2017; Cani, 2018). Moreover, some studies have verified that metformin directly promotes the growth of this bacterium *in vitro* (Wu et al., 2017).

### 4.3 Modulation of SCFAs-producing bacteria by metformin

SCFAs (such as acetic acid, propionic acid and butyric acid) are usually metabolites produced by various SCFAs-producing bacteria in the gut that metabolizes carbohydrates such as dietary fiber (Rau et al., 2018), and they are essential for the health of the gut, the body and even the brain. SCFAs can affect brain function by directly or indirectly regulating the gut-brain axis through immune, vagal, endocrine and other humoral pathways (Dalile et al., 2019). They also suppress appetite as well as regulate energy homeostasis by stimulating the secretion of hormones such as GLP-1 from intestinal L cells (De Silva and Bloom, 2012). There is evidence that metformin can increase the abundance of microbiota which can all produce SCFAs such as *Butyrivibrio*, *Bifidobacterium*, *Megasphaera*, *Prevotella*, and so on (de la Cuesta-Zuluaga et al., 2017).

### 4.4 Regulation of *Lactobacillus* by metformin

*Lactobacillus* is a class of anaerobic or partly anaerobic, non-bacteriophage bacteria belonging to the phylum Firmicutes. The bacteria of this genus have a strong ability to break down sugar, and the main end product is lactic acid, which can protect the intestinal barrier and reduce the inflammatory response. Metformin has been found to increase the abundance of *Lactobacillus* in rodents with HFD (Zhou et al., 2016; Bauer et al., 2018). In recent studies, this trend has also been observed in certain clinical studies in patients with T2DM (Xu et al., 2022).

### 4.5 Regulation of disease-associated bacteria by metformin

One study Forbes et al. (2018) indicated that the gut microbiota of patients with immune-mediated inflammatory diseases such as IBD, ankylosing spondylitis, rheumatoid arthritis and systemic lupus erythematosus differed sharply from normal controls. For example, the abundance of *Intestinibacter* is sensibly higher in the gut of patients with Crohn’s disease (CD). In addition, *Bacteroides fragilis* is the only bacterium that has been proved to beget abscess formation, has a potent virulence factor, and is the most common anaerobic pathogen (Wexler, 2007). Other bacteria associated with the disease are *Clostridioides difficile* [here we use its new nomenclature according to Zheng et al. (2020)], an anaerobic enteric pathogen that can cause severe diarrhea and lead to death (Sandhu and Mcbride, 2018). Metformin declines the number of disease-associated bacteria such as *Intestinibacter* spp., *Bacteroides fragilis*, and *Clostridioides difficile* (de la Cuesta-Zuluaga et al., 2017; Elbere et al., 2018; Bryrup et al., 2019).

## 5 Mechanisms by which metformin acts through regulation of the gut microbiota

### 5.1 Acts by stabilizing the mucosal barrier of the GI tract

The GI mucosal barrier with important physiological roles is a three-dimensional protective structure consisting of mechanical, chemical, immune and biological barriers. Many of the mechanisms of metformin action revolve around stabilization of the GI mucosal barrier. An emphatic increase in the expression of two markers of mucin levels, MUC2 and MUC5 genes, was observed after metformin treatment (Kyriachenko et al., 2019). This suggests that the increased abundance of *A. muciniphila* after metformin administration may be due to the fact that metformin increases the number of goblet cells, providing more substrate for the growth of *A. muciniphila*. Moreover, *A. muciniphila* was discovered to improve HFD-induced intestinal hyperpermeability and also affect intestinal barrier function by the way that upregulates the expression of tight junction and closure proteins (Li et al., 2016). In this way, it decreases the entry of pro-inflammatory lipopolysaccharides (LPS) into the circulation, thus reducing inflammation in the organism. Additionally, Everard et al. (2013). measured the concentration of endocannabinoids (ECs) in the gut of the mice treated with *A. muciniphila* and found that *A. muciniphila* also raised the release of ECs. Increased endocannabinoids inhibit monoacylglycerol lipase (Alhouayek et al., 2011), thereby reducing systemic inflammation, altering intestinal peptide secretion and increasing intestinal mucosal barrier thickness. It is well known that T2DM is a metabolic and inflammatory disease characterized by deteriorating islet function and elevated levels of inflammatory cytokines. Therefore, therapies targeting inflammation can also recover glycemic control in T2DM patients. In a study, the treatment with three different strains of *A. muciniphila* was found to reverse the low-grade chronic inflammatory state in HFD mice to a certain extent, including a significant increase in the gene expression of inflammatory and immunosuppressive factor interleukin (IL)-10, as well as a decrease in the mRNA levels of tumor necrosis factor-α (TNF-α), Monocyte chemoattractant protein-1, and Toll-like receptor 2 (TLR2) (Yang et al., 2020). In terms of alleviating inflammation, *A. muciniphila* also modulates Forkhead box protein 3 (Foxp3) in mouse adipose tissue (Choi et al., 2021), resulting in a diminution of regulatory T cells (Tregs), and anti-inflammatory while indirectly improving glucose homeostasis. In addition, a specific membrane protein, Amuc_1100, exists on the outer membrane of *A. muciniphila*, and many studies have reported that it induces the production of specific cytokines by binding to TLR2 and activating the downstream pathway of TLR2 (Figure 1), which in turn improves the host’s immune homeostasis and intestinal mucosal barrier function (Plovier et al., 2017; Song et al., 2023; Wang et al., 2024), and then improves the blood glucose level. The protein retains its active conformation and functions even after pasteurization (Plovier et al., 2017). Amuc_1100 protein also has the function of lowering blood lipids, which can reduce the content of low-density lipoprotein (LDL) and cholesterol (Lee et al., 2018). Plus, vesicles secreted by *A. muciniphila* reduce the expression of Toll-like receptor 4 (TLR4) (Figure 1), which impacts the nuclear factor-κB (NF-κB) pathway and thus lessens the secretion of the pro-inflammatory factors IL-6, IL-8 (Ottman et al., 2017; Ashrafian et al., 2019).

What’s more, metformin can increase the abundance of *Bifidobacterium* which can promote the growth of gastric mucin, thereby stabilizing the GI mucosa. Cani et al. (2009) identified that after increasing the quantity of *Bifidobacterium* in the intestine, the content of proglucagon mRNA was higher, which promoted the secretion of glucagon-derived peptides. During the course, glucagon-like peptide-2 (GLP-2) enhanced intestinal epithelial cells proliferation and reduced gut permeability, thereby stabilizing the intestinal mucosal barrier.

### 5.2 Acts by facilitating the synthesis of SCFAs

For the gut, SCFAs have important roles in regulating intestinal microbiota balance, maintaining electrolyte balance and improving intestinal immune function. Administration of metformin increases the abundance of SCFAs-producing microbiota (described previously, see Chapter 4.3), thereby increasing the production of SCFAs. The produced SCFAs are partially absorbed rapidly by colonocytes via monocarboxylate transporter protein (MCT), then undergo a series of oxidative reactions, and finally provide energy to the cells in the form of ATP (Dalile et al., 2019). The SCFAs not metabolized in colonocytes enter the portal circulation of the liver through the basolateral membrane and provide substrates for energy metabolism in hepatocytes. SCFAs are also implicated in the biosynthesis of glucose, fatty acids and cholesterol in hepatocytes. After the above metabolism, only a small fraction of SCFAs remains to reach the whole body through the blood circulation (Dalile et al., 2019).

After SCFAs are absorbed in the colon, it works further via action on the G protein-coupled receptors GPR43 and GPR41 (Figure 1). SCFAs stimulate the proliferation of colonic epithelial L cells and are bound to GPR43 above them, increasing intracytoplasmic calcium ion and cyclic adenosine cAMP concentrations and increasing GLP-1 secretion (Chambers et al., 2018; Rodriguez et al., 2018) which inhibits glucose uptake by intestinal wall cells. Activation of GPR41 increases the secretion of intestinal peptide YY (PYY) and decreases the secretion of glucose-dependent insulin-releasing polypeptide (GIP) and growth hormone-releasing peptide, thereby reducing glucose uptake (Hu et al., 2018). GPR43 is distributed in adipose tissue, bone marrow, spleen, pancreas, peripheral blood mononuclear cells, small intestine, and mammary gland, while GPR41 is distributed in various tissues, especially in immune cells. Hence, SCFAs also have beneficial effects on peripheral tissues, controlling substrate metabolism and optimizing systemic insulin sensitivity. For example, in immune cells, SCFAs can inhibit inflammation by binding to GPR41 and GPR43 (Allin et al., 2015; Thorburn et al., 2015). SCFAs also induce browning of white adipose tissue, boost glucose tolerance, control blood glucose levels, and exert anti-obesity and anti-diabetic effects (He et al., 2020).

Moreover, SCFAs are inhibitors of histone deacetylase (HDAC). SCFAs inhibit HDAC of Treg, in turn affecting Treg production (Arpaia et al., 2013; Furusawa et al., 2013). Also, the inhibition of HDAC by SCFAs can further modulate the function of intestinal macrophages (Chang et al., 2014) and dendritic cells (Trompette et al., 2014; Meijnikman et al., 2018) and downregulate LPS-induced pro-inflammatory mediators, such as NO, IL-6, and IL-12, resulting in anti-inflammatory effects. There is also evidence that SCFAs decline the release of TNF-α from neutrophils (Lee et al., 2019), which is also one of the typical pro-inflammatory cytokines that trigger subclinical inflammation.

Notably, SCFAs also play a crucial role in maintaining the Intestinal mucosal barrier integrity. SCFAs can increase transcription of mucin genes (described previously, see Chapter 4.1), then upgrading intestinal barrier function (Willemsen et al., 2003; Gaudier et al., 2004). At the same time, the increased *A. muciniphila* would continue to degrade mucin and produce more SCFAs. A prospective study by Dao et al. (2016) verified the relationship. This study spotted that individuals with higher abundance of *A. muciniphila* had a better metabolic profile, and the concentration of SCFAs in such populations was positively correlated with *A. muciniphila* abundance.

### 5.3 Acts by regulating bile acid metabolism

Bile acids are a class of amphiphilic metabolites produced by hepatocytes and secreted by the bile ducts into the intestine, which have an important emulsifying effect on fat ingested by the body during digestion. The substantial increase in plasma bile acid concentrations (primary, secondary, total and unconjugated bile acid concentrations) with metformin may be explained by the fact that metformin increased the abundance of probiotic bacteria, especially *Lactobacillus*, and thus their bile salt hydrolase (BSH) gene expression. The BSH gene, a gene encoding the production of BSH by the intestinal microbiota, is associated with obesity and T2DM was significantly correlated (Jia et al., 2020). An essential part of cholesterol regulation by gut microbes is achieved by the hydrolysis of cross-linked bile salts by BSH, thus reducing serum cholesterol levels. The de-cross-linked bile acids have low water solubility and are readily secreted into the feces. Due to the loss of fecal bile acids and the decrease in bile salts, the liver increases the conversion of cholesterol to bile salts to maintain bile salt metabolic balance. It has been demonstrated that oral administration of BSH-containing *Lactobacillus* can reduce serum cholesterol levels in animals and humans, especially *Limosilactobacillus fermentum* [here we use its new nomenclature according to Naghmouchi et al. (2020), Zheng et al. (2020), Çiftci and Tuna (2021), Ng et al. (2022)]. However, no studies have revealed a correlation between the presence of specific bacteria and bile acid concentrations after metformin treatment (Wu et al., 2017).

Subsequently, secondary bile acids, key signaling molecules produced by bile acids and gut microbiota metabolism, are bound to membrane receptors (Takeda G protein-coupled receptor 5, TGR5/G protein-coupled bile acid receptor 1, GPBAR1) and nuclear receptors (Farnesoid X Receptor, FXR), which stimulates colonic secretion of GLP-1 and PYY. It plays a role in promoting proliferation and inhibiting apoptosis in pancreatic 𝛽-cells, and directly scales insulin secretion up. Another study (Sun et al., 2020) demonstrated that serum fibroblast growth factor 19 (FGF19) and bile acid levels were simultaneously increased in patients with T2DM early in life. This may be due to the fact that bile acids entering the intestine stimulate intestinal secretion and expression of the gut-derived hormone FGF19 via bile acid-FXR signaling (Mulla et al., 2019), which stimulates hepatic glycogen synthesis in an insulin non-dependent manner, restrains hepatic gluconeogenesis, and exerts hypoglycemic effects. FGF19 also dramatically promotes glucose tolerance (Zhang et al., 2019).

There are other pathways for FXR signaling activation. Sun et al. (2018) showed that metformin treatment altered the metabolism of folate and methionine in mice, which in turn inhibited the growth of *B. fragilis*. The reduction of *B. fragilis* increased the level of Glycoursodeoxycholic acid in the gut, then led to the activation of intestinal FXR signaling. Several studies have implied that FXR activation relieves intestinal inflammatory state in IBD patients, as well as that colonic inflammation in patients with CD and in rodent models of colitis is linked to diminished expression of FXR mRNA (Vavassori et al., 2009). During inflammation in the liver, there is a sustained inhibition of bile salt export pump expression and reduced FXR expression. Hepatic transporter protein function is then decreased, which leads to both an increase in hepatic bile acid chelation and persistent inflammation. Activation of FXR stabilizes the nuclear co-repressor (NcoR) at the NF-κB response element on the IL-1β promoter, while decreasing the expression of TNF-α, IL-1β, IL-2, IL-6 and IFN-γ mRNA, thereby reducing the extent of inflammation (Vavassori et al., 2009).

### 5.4 Acts by modulating gene expression in the intestinal microbiota

In addition to regulating the gene expression of BSH of probiotic bacteria (described previously, see Chapter 4.3), metformin also modulates the protein-coding genes of another microbiota. Wu et al. (2017) discovered that the abundance of *E. coli*, *Bifidobacterium adolescentic*, and *A. muciniphila* was plainly increased in the intestine after metformin administration. They further proceeded transcriptomic analysis of fecal samples and found that some of the protein-coding genes of *A. muciniphila* and *B. wadsworthia* were dramatically regulated by metformin, and most of them encoded metalloproteins or metal transporter proteins. Metal ions (e.g., Ca2+, Mn2+, Zn2+, and Mg2+) play an important role in maintaining the structure and homeostasis of various proteins in microorganisms. And these proteins affect the metabolism of microorganisms (Murphy et al., 1981). Several studies have shown that metallothionein has a negative regulatory role in all kinds of organ tissues, various types (including LPS-related, allergic and oxidative) of inflammation (Inoue et al., 2009; Inoue and Takano, 2013). It has also been indicated that metformin is able to bind to metal ions, thus causing changes in chemical structure (Logie et al., 2012). However, further metabolomic and proteomic studies are still needed to investigate the mechanisms of relevant microbial metabolism-host interactions.

### 5.5 Other effects of metformin

Metformin was found to reduce the number of *C. difficile* in the intestine in several studies (Elbere et al., 2018; Bryrup et al., 2019). *C. difficile* can release toxin A and toxin B (Popoff, 2018), which induce the production of pro-inflammatory cytokines and inflammatory mediators in a variety of cell lines (Zhang and Feng, 2016). And toxin A is able to trigger IL-8 secretion by human intestinal cells (He et al., 2002). It has also been reported that prostaglandin E2 (PGE2) secretion and cyclooxygenase-2 (COX-2) expression were significantly increased in colon cells exposed to toxin A either *in vivo* or *in vitro* (Kim et al., 2007). Therefore, metformin is able to decrease the release of pro-inflammatory factors by a reduction in the number of opportunistic pathogenic bacteria.

## 6 Summary and outlook

Metformin has been used globally for more than 60 years as the drug of choice for the treatment of T2DM. In clinical applications, in addition to treating T2DM, metformin has been found to be effective in certain diseases, such as obesity, cardiovascular disease, IBD, and other inflammation-related diseases. In addition, metformin has received much attention for its anti-tumor and anti-aging effects. Even recent literature provides evidence that metformin slows the development of COVID-19. Some people may experience side effects after taking metformin, with the main symptoms being gastrointestinal distress and, in rare cases, lactic acidosis (Sanchez-Rangel and Inzucchi, 2017). However, the mechanisms involved, whether therapeutic or side effects, are not fully understood. Previously, it was believed that metformin targets the liver and exerts its hypoglycemic effect through AMPK-dependent and non-AMPK-dependent pathways. However, this is not sufficient to explain the mechanism by which metformin achieves its broad therapeutic effects. A number of studies have provided evidence that the gut is the site of metformin’s wide-ranging effects, and found that gut microbiota may be an important “target” in this regard. However, differences in study subjects and experimental designs, especially the heterogeneity of the gut microbiota itself, have made it difficult to study the modulatory effects of metformin on the gut microbiota. The results of changes in the diversity and abundance of the human gut microbiota after metformin treatment are not consistent. Changes in the abundance of certain microbiota were also not identical across studies, and it is even possible to draw opposite conclusions. This highlights the importance of the need for further research to understand the underlying mechanisms of these changes. Nonetheless, metformin use has been associated with changes in the abundance of specific bacterial genera in the gut microbiota, and this has centered on the enrichment of *A. muciniphila* and bacteria that can produce SCFAs. By enriching these bacteria to stabilize the GI mucosal barrier as well as to regulate bile acid metabolism, metformin exerts a role in regulating glucose metabolism and lipid metabolism, which in turn maintains glucose homeostasis and improves the inflammatory state. In addition, with the increased interest of researchers in multi-omics studies, transcriptomic and metabolomic studies on the gut microbiota have increased. It was gradually found that metformin can also regulate the expression of certain genes in the gut microbiota, such as BSH genes and metalloproteins genes. However, the intestinal microbiota is very complex and it is challenging to study the mechanism of action of metformin. The mechanism by which metformin acts by regulating the gut microbiota needs to be further clarified.

## Author contributions

YW: Investigation, Writing – original draft. XJ: Writing – original draft. BC: Writing – review & editing, Conceptualization, Supervision.
